# Integrated optimization of spatiotemporal resources at the intersection for delay minimization using genetic algorithm

**DOI:** 10.1371/journal.pone.0339519

**Published:** 2026-01-23

**Authors:** Zhen Yang, Lan Wu, Gen Li, Yichao Xu, Wenhao Liu

**Affiliations:** 1 College of Automobile and Traffic Engineering, Nanjing Forestry University, Nanjing, China; Southwest Jiaotong University, CHINA

## Abstract

Integrated optimization of spatiotemporal resources at the intersection (IOSTRI) is crucial for traffic signal control, where both the lane allocation and signal control plans are optimized in a unified framework. This paper addresses the IOSTRI problem with delay minimization, formulating it as a binary mixed-integer nonlinear program (BMINLP) model that fully incorporates all possible uses of shared lanes and lane utilization adjustments. A genetic algorithm tailored to the model’s characteristics is designed, where four modules named lane converter, signal plan converter, flow calculation function and delay calculation function are used to calculate the fitness of each solution. Numerical results show the proposed model and algorithm’s ability to adapt to diverse traffic flow distribution patterns. High-quality solutions are obtained within 40–55 seconds, representing a significant improvement over previous studies and satisfying the requirements for real-time adaptive control of a single intersection.

## 1. Introduction

With the rapid growth of traffic demand, urban road networks are becoming increasingly crowded. Many cities face serious problems of traffic congestion. Intersections are often seen as bottlenecks in the road network, where conflicting traffic from different directions causes delays and stops. Therefore, effective management and control of intersections not only concern with the safety and efficiency of road network, but also one of the viable solutions to alleviating traffic congestion [1,2]. To ensure the capacity of intersection, sufficient lanes need to be allocated to accommodate traffic from different directions, so that the lane allocation or lane distribution can be regarded as the spatial resource of intersection. On the other hand, to address conflicts between traffic from different directions, a reasonable signal control plan (including phase structure and signal timing) needs to be implemented to arrange each traffic stream in a certain period. Proper designed signal timing also enhances the efficiency of intersection and reduces vehicle travel time. Therefore, the signal control plan can be regarded as the temporal resource of intersection.

The integrated optimization of spatio-temporal resources at the intersection (IOSTRI) is an important issue in the research field of traffic signal control, where both lane settings and signal control parameters are regarded as decision variables [3]. The problem of IOSTRI have both theorical and practical meanings. At the theoretical level, given the traffic demand and geometric dimensions at the intersection, various models with different optimization objectives and complexities have been proposed, along with algorithms developed to obtain the global optimal solutions or converged ones. At the practical level, the outcomes of the IOSTRI model can be utilized by the traffic management sector to enhance the lane configuration and signal control scheme of a real intersection.

According to the optimization objectives, the problem of IOSTRI can be divided into two categories: 1) cycle length minimization or capacity maximization; 2) delay minimization. For optimization objective of cycle length minimization or capacity maximization, the models are typically formulated as binary mixed integer linear program (BMILP). Global optimal solution can be obtained by branch and bound algorithm [3]. In this branch field, Wong et al. (2003) did a pioneering work by integrating lane markings, signal settings, traffic and pedestrian movements in a unified framework. The integer variables include the permitted movements on approach lanes and successor functions to specify the order of signal displays. The continuous variables include the assigned lane flows, common flow multiplier, cycle length, starts and durations of green for each traffic movement, and pedestrian crossing time [3]. Later, Wong et al. (2005, 2006) considered the time-varying characteristic of traffic demand and introduced multi-period decision variables into the model [4,5]. Wong and Heydecker (2011) did an extension work to relax the number of approach lanes within intersection arms as new integer variables [6]. In subsequent research, Liu and Wong (2017) refined the capacity maximization model to make it satisfy the spatial queue requirements for all approach lanes, including the short lanes [7]. Wong and Lee (2020) further considered the factor of queuing space and proposed new constraints to control the effective red durations, thus prevent queue spillback and residual queues [8]. Wong and Liu (2017, 2019) extended the capacity maximization model to signal-controlled road network with origin and destination (OD) demand flows as inputs, and incorporated green band maximization in the objective function [9,10]. In recent years, other scholars also made important contributions to this branch. Tang et al. (2019) proposed a capacity maximization model with left turn prohibition. The model is then combined with stochastic user equilibrium model to minimize total travel time on the network [11]. In subsequent research, Tang and Sohr (2020) proposed a capacity maximization model that can deal with the incompatible movements merging at the same destination arm [12]. Zhao and Ma (2021) established a model to maximize the capacity of the intersection under the tandem control and exit-lanes for left-turn traffic, where the mixed-usage lane, lane allocation and signal timings can be integrated and optimized [13]. Zhao et al. (2022) built an integrated model to optimize the geometric layout and signal control scheme for intersections with work zones. The lanes adjacent to work zone can be used dynamically as approach and exit lanes during a signal cycle [14]. Dai et al. (2024) proposed a joint method for optimizing spatiotemporal resources at intersections under mixed-autonomy traffic conditions. The method can accommodate to substantial traffic demand fluctuations and have no significant sensitivity to the connected and automated vehicle (CAV) market penetration rate [15]. Shen et al. (2025) proposed an integrated model that incorporates lane allocation, signal timings, and various types of shared right-turn lane control strategies. The permitted right-turn movement or phase under pedestrian interference was taken into consideration [16].

For IOSTRI problem with the objective of delay minimization, since the delay function is non-linear (e.g., as described in the delay calculation procedure of the Highway Capacity Manual), the models are typically formulated as binary mixed integer non-linear program (BMINLP). Compare to BMILP, it is more difficult to obtain a global optimal solution of BMINLP [17,18]. However, since delay measurement is directly related to the performance of the intersection, the solutions of BMINLP hold more practical significance than those of BMILP. In this branch field, Wong et al. (2003) proposed a classical cutting plane algorithm and a heuristic line search algorithm to solve the delay minimization model. The cutting plane algorithm takes very long time to meet the convergence criterion, whereas the heuristic line search algorithm is based on cycle length minimization and capacity maximization models, thus has higher computational efficiency. However, the computing time is still as long as 40 minutes [17,18]. In subsequent research, Wong and Lee (2012) improved the cutting plane algorithm by introducing a 2D convergence density stop criterion. This new criterion makes it easier to obtain a converged solution [19].

In recent years, other scholars have applied new methodologies to solve the delay minimization model, or developed similar models in a new context. Ratrout and Assi (2021) proposed a non-liner model to collectively optimize the signal timing plan, lane allocation and phase scheme [20]. However, the model is only based on two types of single ring phase scheme. In subsequent research, Assi et al. (2023) developed the brute force-based model to find the optimal lane assignments and cycle lengths for a wide range of traffic flow combinations with the objective of delay minimization. A substantial synthetic dataset was produced based on these optimal solutions. The dataset was subsequently used to train a deep learning model to predict the optimal lane assignment and cycle length for specific traffic flow combinations [21]. However, this method requires that the intersection geometry remains consistent, thereby limiting its transferability. Hao and Jin (2023) developed a breadth first search algorithm and a branch pruning strategy to improve the solving process and computation efficiency of the delay minimization model, respectively [22]. However, their method requires the enumeration of all feasible lane allocation schemes, and it still took over 80 minutes to obtain the optimal solution for a typical intersection with four lanes at each arm. Huang et al. (2024) developed a two-stage delay minimization model considering the fluctuation of traffic demand, and level-of-service (LOS) reliability is introduced to reduce the computational difficulty [23]. However, the model allows the through traffic to conflict with left-turn traffic, which is unrealistic, and the computation time is still quite long, requiring one hour for each iteration. Wang et al. (2024) proposed a delay minimization model that integrates dynamic lane assignment and signal timing plan, also the impact of lane switching is considered [24]. However, their method primarily focuses on variable lanes, while the functions of most lanes remain fixed. Huang et al. (2025) developed a two-layer framework for integrated optimization of lane allocation and signal timing plans at isolated intersections, and a dynamic control scheme was also proposed to regulate the frequency of lane switching [25]. However, the lane allocation and signal timing modules are at different levels, which may negatively affect the optimization results.

Some researches also dealt with IOSTRI problem with exclusive bus lanes. Zhao and Zhou (2018) developed a delay minimization model in which the exclusive bus lane at the exit can be dynamically used for the left-turn buses and opposing through buses [26]. However, only the uniform delay is considered in the objective function to make the model turn into the convex quadratic programming. Shi et al. (2020) presented a delay minimization model for the integrated design of exclusive bus lanes, passenger car lane allocations, and the signal plan with passive transit priority [27]. However, the lane function is limited to exclusive lanes, and shared lane use is not considered.

From the above research, we can see that for IOSTRI problem with the objective of delay minimization, there is still a lack of effective algorithms to obtain the optimal solution (or a satisfactory near-optimal one) in a reasonable time. As advanced traffic control systems are widely applied in many cities, the computation time of delay minimization model should satisfy the need of real-time adaptive control. Also, very few researches have fully incorporated the use of shared lanes at the intersection, which is an important part of lane allocation optimization. The proper use of shared lanes can make the traffic flow more equally distributed, thereby increase the intersection capacity. Moreover, when more than one exclusive lane is assigned for a certain movement (e.g., three through lanes at one arm), the traffic for that movement tends to be unequally distributed across these lanes, resulting in a reduction in the saturation flow rate. In other words, the saturation flow rate for a specific movement will change as the number of lanes increases. Existing research ignored this factor, assuming that the saturation flow rate is not sensitive to changes in the number of lanes. The Highway Capacity Manual (HCM) [28] also includes a lane utilization adjustment factor for this situation, indicating that the impact of lane utilization on the saturation flow rate should be considered during model development. Therefore, this paper will address the drawbacks of existing researches and conduct a more comprehensive study of IOSTRI problem with delay minimization.

## 2. Research objective

With regard to the shortcomings of existing literature, this study proposes an integrated optimization model for the IOSTRI problem, with delay minimization as its objective. The combinations of shared lanes and the use of lane utilization adjustment factor are fully incorporated. The model is also based on the dual-ring phase structure, which can illustrate the conflicts between phases at the intersection. A genetic algorithm is then designed to solve the integrated optimization model to achieve a converged solution within a reasonable time.

## 3. Model establishment

By fully incorporating the combinations of shared lanes and introducing the lane utilization adjustment factor, this section establishes a BMINLP model to optimize the lane allocation and signal control scheme within an integrated framework at the intersection.

### 3.1. Definition of index variables

**Fig 1:**
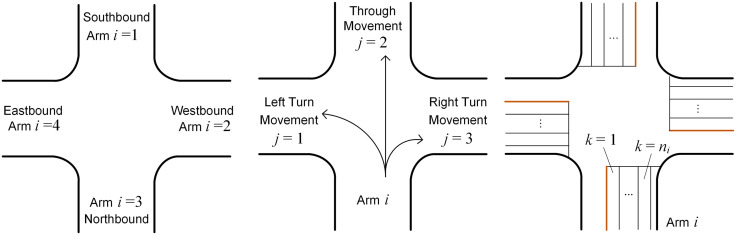
The definition of index variables. (a) Global index of intersection arms. (b) Local index of vehicle movements. (c) Local index of approach lanes.

For a typical intersection with four arms, let *A* be the set of intersection arms and *A* = {1, 2, 3, 4}. Let *i* be the global index of intersection arms and *i* ∈ *A*. The value of *i* and its corresponding direction are illustrated in Fig 1(a), where the arms are numbered in a clockwise direction with the southbound arm numbered as *i* = 1 [3].

Let *D* be the set of vehicle movements at the intersection and *D* = {1, 2, 3}. Let *j* be the local index of vehicle movements and *j* ∈ *D*. The value of *j* and its corresponding movement are illustrated in Fig 1(b), where the immediate left arm with respect to arm *i* is numbered as *j* = 1, and the other arms are numbered consecutively in a clockwise direction. A movement or a phase can then be represented as (*i*, *j*) which means a direction *j* from arm *i*. The local index *j* can be converted into global index *i* by using Equation (1):


$$𝛤(i,j)=\left\{ \;\;\;\begin{array}{c} i+j,\;i+j\leq 4\\ i+j-4,\;i+j>4 \end{array}\right.\;\;\;\;,\;\forall i\in A,j\in D$$ (1)

where *Γ* (*i*, *j*) is the corresponding global index value of (*i*, *j*) [3]. For instance, if a vehicle turns left (*j* = 1) from arm *i* = 4, the destination arm is *i* = 1 according to Equation (1).

Let *K*_*i*_ be the set of approach lanes at the intersection arm *i*. Let *k* be the local index of approach lanes and *k* ∈ *K*_*i*_. The value of *k* and its corresponding lane at arm *i* are illustrated in Fig 1(c), where the leftmost lane is numbered as *k* = 1, and the rightmost one is numbered as *k* = *n*_*i*_ (*n*_*i*_ is the total number of lanes at arm *i*). The other lanes are numbered consecutively from left to right.

### 3.2. The phase scheme

**Fig 2:**
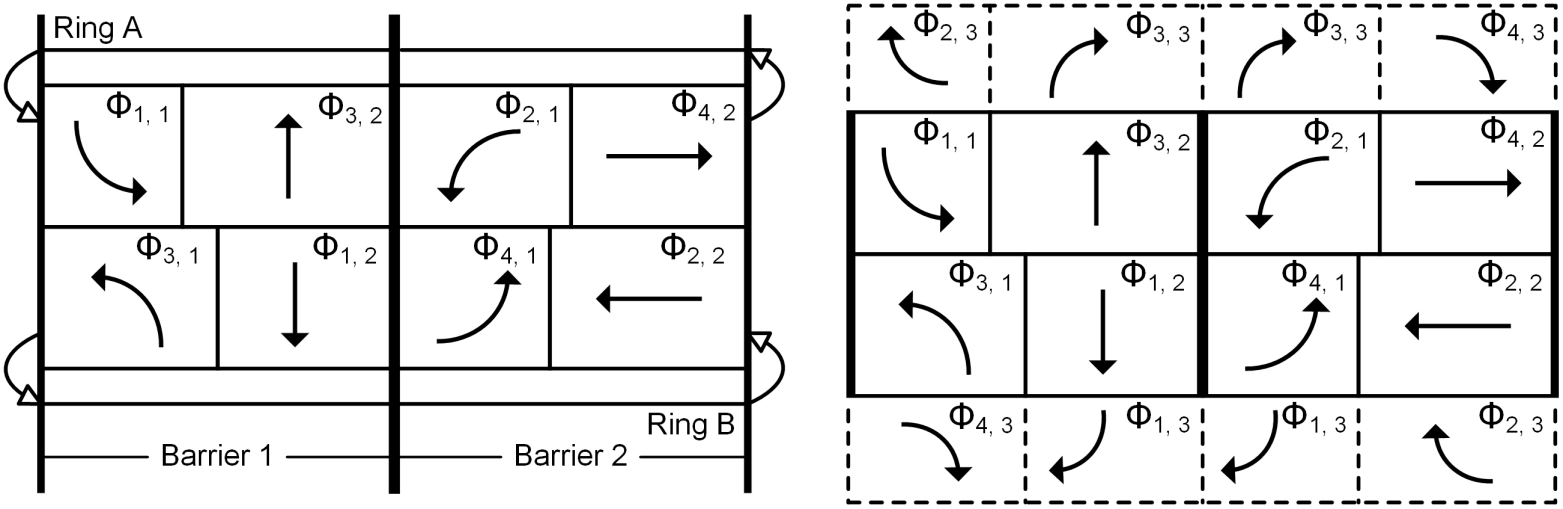
The dual-ring phase scheme. (a) The organization of left-turn and through phases. (b) The organization of right-turn phases.

The dual-ring phase scheme, as specified by the National Electrical Manufacturing Association (NEMA) Standards [29], is employed in the model. As illustrated in Fig 2, this phase scheme adopts the concepts of ring and barrier to organize the conflicting phases at the intersection. In Fig 2, *Φ*_*i*, *j*_ (*i* ∈ *A*, *j* ∈ *D*) is equal to the duration of phase (*i*, *j*) splits divided by the cycle length, where the duration of each phase split includes the green and clearance intervals. Fig 2(a) shows the organization of left-turn and through phases, where two phases located in the same barrier and different rings can operate concurrently (For example, phase (1, 1) and (3, 1), phase (1, 1) and (1, 2)); otherwise, the phases can only operate one at a time. Based on Fig 2(a), Fig 2(b) adds the right-turn phases, which are presented by dashed border. Each right-turn phase can overlap with the through one at the same arm, the left-turn one at the right arm, or both (For example, phase (3, 3) can overlap with phase (3, 2), phase (2, 1), or both).

### 3.3. The constraints

With the index variables and phase scheme stated above, this section describes the constraints of the integrated optimization model, including lane allocation, lane utilization, ring-barrier structure, phase duration, flow volume, flow ratio and saturation flow rate.

#### 3.3.1. Lane allocation.

Denote binary variable *x*_*i*, *j*, *k*_ as the movement permission decision variable, which presents whether direction *j* is placed on lane *k* at arm *i*, where *x*_*i*, *j*, *k*_ = 1 means direction *j* is placed on lane *k* at arm *i*, and *x*_*i*, *j*, *k*_ = 0 means otherwise. It is obvious that at least one lane should be provided for direction *j* at arm *i*, so that:


$$\sum_{k}x_{i,j,k}\geq 1,\;\forall i\in A,\;j\in D$$ (2)

In addition, since there are a total of three directions (left-turn, through, and right-turn) at arm *i*, certain conditions are needed to constrain the number of lanes for each direction. These can be expressed as the follow:


$$n_{i,1}+n_{i,3}\leq \left\{ \;\;\;\begin{array}{c} n_{i}-1,\;\Delta_{i,LT}=\Delta_{i,RT}=0\\ n_{i},\;\Delta_{i,LT}+\Delta_{i,RT}=1\\ n_{i}+1,\;\Delta_{i,LT}=\Delta_{i,RT}=1 \end{array}\right.\;\;\;\;\;,\;\forall i\in A$$ (3)

where *n*_*i*, *j*_ is the number of lanes for movement (*i*, *j*); Δ_*i*, LT_ and Δ_*i*, RT_ are decision variables of shared lane use at arm *i*. For Δ_*i*, LT_, if Δ_*i*, LT_ = 1, the through movement is shared with left-turn one at arm *i*; if Δ_*i*, LT_ = 0, otherwise. For Δ_*i*, RT_, if Δ_*i*, RT_ = 1, the through movement is shared with right-turn one at arm *i*; if Δ_*i*, RT_ = 0, otherwise. Note that for each type of shared lane (shared left-through lane, shared through-right lane, and shared left-through-right lane), a maximum of one lane can be employed at arm *i*. If a shared left-through-right lane is employed, the other types of shared lanes are not allowed. Therefore, Equation (3) means if no shared lane (only exclusive lanes) is employed at arm *i*, the total number of left and right-turn lanes should not exceed *n*_*i*_ −1, otherwise there will be no sufficient space for through lanes; If shared left-through or through-right lane is employed at arm *i*, the total number of lanes with left and right-turn movements should not exceed *n*_*i*_ (where being equal to *n*_*i*_ means there is no exclusive through lane); If through movement is shared with both left and right-turn ones at arm *i*, the total number of lanes with left and right-turn movements should not exceed *n*_*i*_* *+ 1 (where being equal to *n*_*i* _+ 1 means a shared left-through-right lane is adopted).

Finally, for any movement (*i*, *j*), the total number of approach lanes should not exceed the number of corresponding exit lanes. This can be expressed as the follow:


$$\sum_{k}x_{i,j,k}\leq E_{\Gamma (i,j)},\;\forall i\in A,\;j\in D$$ (4)

where *E*_*Γ* (*i*, *j*)_ is the number of exit lanes for movement (*i*, *j*).

#### 3.3.2. Lane utilization.

According to HCM, when more than one lane is provided for a certain movement, the vehicles tend to distribute uniformly on these lanes, resulting in the reduction of capacity for this movement. Therefore, the lane utilization adjustment factor is introduced by HCM to adjust the saturation flow rate in such a situation. The values of lane utilization adjustment factors for different types of lanes are shown in Table 1.

**Table 1:** The lane utilization adjustment factor specified by HCM.

Lane type	Number of lanes	Lane utilization adjustment factor
Exclusive through lane or shared lane	1	1.000
	2	0.952
	≥3	0.908 (or observed in field)
Exclusive left-turn lane	1	1.000
	≥2	0.971 (or observed in field)
Exclusive right-turn lane	1	1.000
	≥2	0.885 (or observed in field)

By introducing the variable *f*_*i*, *j*_ as the value of lane utilization adjustment factor for movement (*i*, *j*), Table 1 can be converted into the following constraints:


$$f_{i,1}=\left\{ \;\;\;\begin{array}{c} 1,\;n_{i,1}-\Delta_{i,LT}\leq 1\\ 0.971,\;n_{i,1}-\Delta_{i,LT}>1 \end{array}\right.\;\;\;\;\;,\;\forall i\in A$$ (5)

$$f_{i,2}=\left\{ \;\;\;\begin{array}{c} 1,\;n_{i,2}=1\\ 0.952,\;n_{i,2}=2\\ 0.908,\;n_{i,2}\geq 3 \end{array}\right.\;\;\;\;,\;\forall i\in A$$ (6)

$$f_{i,3}=\left\{ \;\;\;\begin{array}{c} 1,\;n_{i,3}-\Delta_{i,RT}\leq 1\\ 0.885,\;n_{i,3}-\Delta_{i,RT}>1 \end{array}\;\;\;\;\;\right.,\;\forall i\in A$$ (7)

The saturation flow rate of each movement can then be adjusted according to Equation (8):


$$S_{i,j,f}=S_{i,j}\cdot f_{i,j},\;\forall i\in A,\;j\in D$$ (8)

where *S*_*i*, *j*_ refers to the single-lane saturation flow rate of movement (*i*, *j*) (veh/h). *S*_*i*, *j*, f_ refers to the actual saturation flow rate of movement (*i*, *j*) after accounting for the lane utilization adjustment factor (veh/h).

#### 3.3.3. Ring-barrier structure and phase duration.

According to the dual-ring phase scheme illustrated in Fig 2, in each barrier, the duration of each ring should be equal. Besides, the duration of each ring should be equal to the cycle length. These can be expressed by the following constraints:


$${\mathrm{\backslash itPhi}}_{i,2}+{\mathrm{\backslash itPhi}}_{\mathrm{\backslash itGamma}(i,2),1}={\mathrm{\backslash itPhi}}_{i,1}+{\mathrm{\backslash itPhi}}_{\mathrm{\backslash itGamma}(i,2),2},\;\forall i\in \{1,\;2\}$$ (9)

$${\mathrm{\backslash itPhi}}_{1,2}+{\mathrm{\backslash itPhi}}_{3,1}+{\mathrm{\backslash itPhi}}_{2,2}+{\mathrm{\backslash itPhi}}_{4,1}=1$$ (10)

For each phase, there should be a minimum duration even if it serves very little traffic demand. That’s because if the phase duration is too short, the driver will not have enough time to react to the signal, which could lead to safety issues. Therefore, each phase should satisfy the following constraint:


$${\mathrm{\backslash itPhi}}_{i,j}\geq T_{i,j,\min}\cdot \zeta ,\;\forall i\in A,\;j\in D$$ (11)

where *T*_*i*, *j*, min_ is the minimum duration of phase (*i*, *j*) (s), and is equal to minimum green time plus the clearance interval; *ζ* is the reciprocal of cycle length (s^-1^). For through phase (like phase (1, 2) in Fig 2), not only should the minimum duration be met, but the pedestrian crossing demand must also be satisfied, as pedestrians typically cross the road during the parallel through phase. This constraint can be expressed as:


$${\mathrm{\backslash itPhi}}_{i,2}\geq P_{i,2}\cdot \zeta ,\;\forall i\in A$$ (12)

where *P*_*i*, 2_ is the time required for pedestrians to cross the road during the parallel through phase (s). It can be calculated by *P*_*i*, 2_ = 7 + *L*_*i*, 2_/ *v*_p_, where the constant number ‘7’ is the duration of pedestrians’ WALK indication (s) [30]; *L*_*i*, 2_ is the length of crosswalk parallel with through phase (*i*, 2) (m); *v*_p_ is pedestrians’ 15th percentile crossing speed (m/s) and can be taken as 1.2m/s in the absence of data.

When the left-turn movement is shared with the through one at arm *i*, the durations of the left-turn and through phases are set to be identical at arm *i*, and the two phases operate simultaneously. This can be expressed as:


$${𝛷}_{i,1}=\left\{ \;\;\;\begin{array}{c} {𝛷}_{i,2},\;\Delta_{i,LT}=1\\ {𝛷}_{i,1},\;\Delta_{i,LT}=0 \end{array}\right.\;\;\;\;,\;\forall i\in A$$ (13)

**Fig 3:**
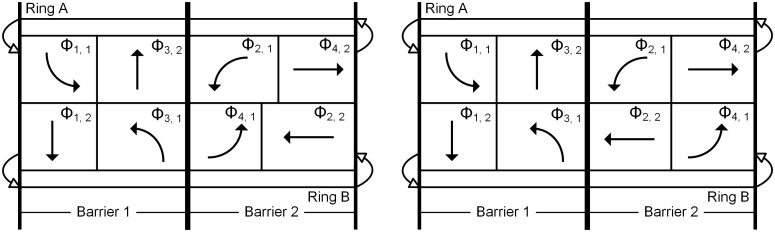
The dual-ring phase scheme for shared left-turn and through movements. (a) one barrier. (b) two barriers.

On the other side, considering the constraint of the barrier (as shown in Equation (9)), the durations of the left-turn and through phases at the opposite arm (*Γ* (*i*, 2)) are also identical. In this situation, the dual-ring phase scheme (as shown in Fig 2) will transition to that shown in Fig 3. It can be seen that the phase sequence in Fig 3 differs from that in Fig 2. Considering that, under the control of a single intersection, the phase sequence does not affect the average vehicle delay, the model does not include constraints related to the phase sequence.

For the right-turn movement, if it operates entirely on exclusive lanes at arm *i*, the right-turn phase can overlap with both the through one at arm *i* and the compatible left-turn one at arm *Γ* (*i*, 3); Otherwise, the right-turn phase can only overlap with the through one. This can be expressed as the follow:


$${𝛷}_{i,3}=\left\{ \;\;\;\begin{array}{c} {𝛷}_{i,2}+{𝛷}_{\Gamma (i,3),1},\;\Delta_{i,RT}=0\\ {𝛷}_{i,2},\;\Delta_{i,RT}=1 \end{array}\right.\;\;\;\;\;,\;\forall i\in A$$ (14)

Additionally, the “right-turn-on-red (RTOR)” for right-turn movements is not utilized in this paper. This is because, under RTOR, the right-turn vehicles are allowed to proceed through the intersection at any time, which increases conflicts with through vehicles. This conflict becomes more serious when there are insufficient exit lanes.

#### 3.3.4. Flow volume, flow ratio and saturation flow rate.

**Fig 4:**
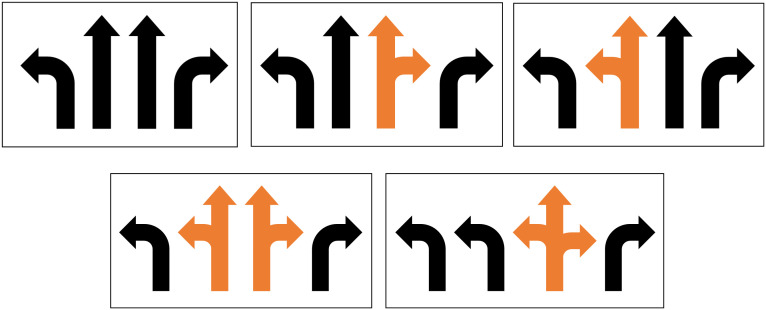
The situations of lane function configurations. (a) Exclusive lanes only. (b) Shared through-right lane included. (c) Shared left-through lane included. (d) Both shared left-through and through-right lanes included. (e) Shared left-through-right lane included.

When the lane allocation for an intersection arm is determined, incoming traffic will be distributed according to the lane function configuration. This section will discuss the situations of lane function configurations (as shown in Fig 4) to calculate traffic flow volume, flow ratio and saturation flow rate on each lane.

▪ **Situation **Ⅰ****: exclusive lanes only

As shown in Fig 4(a), when only exclusive lanes are employed at arm *i*, after lane utilization factor is applied to adjust the saturation flow rate, the traffic flow in each direction is supposed to be equally distributed on the corresponding exclusive lanes. The flow ratio on each lane can be calculated by using Equation (15):


$$y_{i,k}=\left\{ \;\;\;\;\begin{array}{c} \frac{q_{i,1}}{S_{i,1,f}\cdot n_{i,1}},\;x_{i,1,k}=1\\ \frac{q_{i,2}}{S_{i,2,f}\cdot n_{i,2}},\;x_{i,2,k}=1\\ \frac{q_{i,3}}{S_{i,3,f}\cdot n_{i,3}},\;x_{i,3,k}=1 \end{array}\right.,\;\forall i\in A,\;k\in K_{i};\;\Delta_{i,RT}=\;\Delta_{i,LT}=0$$ (15)

where *q*_*i*, *j*_ is the flow volume of movement (*i*, *j*); *y*_*i*, *k*_ is the flow ratio on lane *k* at arm *i*.

▪ **Situation **Ⅱ**:** shared through-right lane included

For this situation (as shown in Fig 4(b)), it is assumed that the through and right-turn flows are distributed in such a way that the flow ratio on each exclusive through lane, shared through-right lane and exclusive right lane is the same [28]. This assumption is also made after the saturation flow rate has been adjusted by the lane utilization factor. The flow ratio *y*_*i*, *k*_ for this situation of lane configuration can be calculated by using Equation (16):


$$y_{i,k}=\left\{ \;\;\;\;\begin{array}{c} \frac{q_{i,1}}{S_{i,1,f}\cdot n_{i,1}},\;x_{i,1,k}=1\mathrm{\backslash vspace}2mm\\ \frac{q_{i,2}/S_{i,2,f}+q_{i,3}/S_{i,3,f}}{n_{i,2}+n_{i,3}-1},\;x_{i,2,k}=1\;\cup x_{i,3,k}=1 \end{array}\right.\;\;\;\;\;,\;\forall i\in A,\;k\in K_{i};\;\Delta_{i,RT}=1,\;\Delta_{i,LT}=0$$ (16)

For shared through-right lane, there are two directions of traffic flow operating on it, which makes its flow volume and saturation flow rate different from those of exclusive lane. After *y*_*i*, *k*_ is obtained, the through and right-turn flow volumes on shared through-right lane can be derived from the volumes on the exclusive lane, as shown in Equations (17) and (18):


$$q_{i,trt}=q_{i,2}-y_{i,tr}\cdot S_{i,2,f}\cdot (n_{i,2}-1),\;\forall i\in A;\;\Delta_{i,RT}=1,\;\Delta_{i,LT}=0$$ (17)

$$q_{i,trr}=q_{i,3}-y_{i,tr}\cdot S_{i,3,f}\cdot (n_{i,3}-1),\;\forall i\in A;\;\Delta_{i,RT}=1,\;\Delta_{i,LT}=0$$ (18)

where *q*_*i*, trt_ and *q*_*i*, trr_ are the flow volumes of through and right-turn traffic on shared through-right lane (veh/h), respectively; *y*_*i*, tr_ is the flow ratio of shared through-right lane. Subsequently, the saturation flow rate of shared through-right lane *S*_*i*, tr_ (veh/h) can be calculated as the follow:


$$S_{i,tr}=(q_{i,trt}+q_{i,trr})/y_{i,tr},\;\forall i\in A;\;\Delta_{i,RT}=1,\;\Delta_{i,LT}=0$$ (19)

▪ **Situation **Ⅲ**:** shared left-through lane included

This situation of lane configuration (as shown in Fig 4(c)) is similar to Situation Ⅱ. The flow ratio on each exclusive left lane, shared left-through lane and exclusive through lane is assumed to be the same, so that *y*_*i*, *k*_ can be calculated by using Equation (20):


$$y_{i,k}=\left\{ \;\;\;\begin{array}{c} \frac{q_{i,1}/S_{i,1,f}+q_{i,2}/S_{i,2,f}}{n_{i,1}+n_{i,2}-1},\;x_{i,1,k}=1\cup x_{i,2,k}=1\\ \frac{q_{i,3}}{S_{i,3,f}\cdot n_{i,3}},\;x_{i,3,k}=1 \end{array}\right.\;\;\;\;\;\;,\;\forall i\in A,\;k\in K_{i};\;\Delta_{i,RT}=0,\;\Delta_{i,LT}=1$$ (20)

After *y*_*i*, *k*_ is obtained, the left-turn and through flow volumes on shared left-through lane can be calculated as the follows:


$$q_{i,ltl}=q_{i,1}-y_{i,lt}\cdot S_{i,1,f}\cdot (n_{i,1}-1),\;\forall i\in A;\;\Delta_{i,RT}=0,\;\Delta_{i,LT}=1$$ (21)

$$q_{i,ltt}=q_{i,2}-y_{i,lt}\cdot S_{i,2,f}\cdot (n_{i,2}-1),\;\forall i\in A;\;\Delta_{i,RT}=0,\;\Delta_{i,LT}=1$$ (22)

where *q*_*i*, ltl_ and *q*_*i*, ltt_ are the flow volumes of left-turn and through traffic on shared left-through lane (veh/h), respectively; *y*_*i*, lt_ is the flow ratio of shared through-right lane. Subsequently, the saturation flow rate of shared left-through lane *S*_*i*, lt_ (veh/h) can be calculated as the follow:


$$S_{i,lt}=(q_{i,ltl}+q_{i,ltt})/y_{i,lt},\;\forall i\in A;\;\Delta_{i,RT}=0,\;\Delta_{i,LT}=1$$ (23)

▪ **Situation **Ⅳ**:** both shared left-through and through-right lanes included, or shared left-through-right lane included

For this situation (as shown in Fig 4(d) and Fig 4(e)), it is assumed that the flow ratios of all lanes are equal, and *y*_*i*, *k*_ can be calculated by using Equation (24):


$$y_{i,k}=\sum_{j=1}^{3}\frac{q_{i,j}}{S_{i,j,f}}/\sum_{j=1}^{3}n_{i,j},\;\forall i\in A,\;k\in K_{i};\;\Delta_{i,RT}=1,\;\Delta_{i,LT}=1$$ (24)

After *y*_*i*, *k*_ is obtained, if shared left-through and through-right lanes are employed separately at arm *i* (as shown in Fig 4(d)), the flow volumes on each shared lane, and the saturation flow rate of each shared lane can be calculated similarly to those of Situations Ⅱ and Ⅲ. If a single shared left-through-right lane is employed (as shown in Fig 4(e)), all through flow volumes will be on this shared lane. Then the saturation flow rate of shared left-through-right lane *S*_*i*, ltr_ (veh/h) can be calculated as the follow:


$$S_{i,ltr}=(q_{i,ltrl}+q_{i,\text{2}}+q_{i,ltrr})/y_{i,ltr},\;\forall i\in A;\;\Delta_{i,RT}=1,\;\Delta_{i,LT}=1$$ (25)

where *q*_*i*, ltrl_ and *q*_*i*, ltrr_ are the flow volumes of left and right-turn traffic on shared left-through-right lane (veh/h), respectively, and can be calculated similarly to those of Situations Ⅱ and Ⅲ; *y*_*i*, ltr_ is the flow ratio of shared left-through-right lane, and is equal to *y*_*i*, *k*_ for this situation of lane configuration.

### 3.4. The objective function

As this paper deals with IOSTRI problem with the objective of delay minimization, the objective function is defined as the minimization of average vehicle delay at the whole intersection, which can be expressed in Equation (26):


$$\min\;z=\sum_{i}\sum_{k}(d_{i,k,1}+d_{i,k,2})\cdot q_{i,k}/\sum_{i}\sum_{k}q_{i,k}$$ (26)

where *d*_*i*, *k*, 1_ and *d*_*i*, *k*, 2_ are the uniform delay (s) and incremental delay (s) for lane *k* at arm *i*, respectively. The calculations of *d*_*i*, *k*, 1_ and *d*_*i*, *k*, 2_ are shown in Equations (27) and (28), respectively:


$$d_{i,k,1}=\frac{0.5\cdot C\cdot {\left(1-\lambda_{i,k}\right)}^{2}}{1-[\min(1,x_{i,k})\cdot \lambda_{i,k}]}$$ (27)

$$d_{i,k,2}=900\cdot T_{a}\cdot [(x_{i,k}-1)+\sqrt{{\left(x_{i,k}-1\right)}^{2}+\frac{4\cdot x_{i,k}}{Q_{i,k}\cdot T_{a}}}]$$ (28)

where *C* is the cycle length of the intersection (s), and is equal to 1/*ζ*; *g*_*i*, *k*_, *x*_*i*, *k*_ and *Q*_*i*, *k*_ are the effective green time (s), degree of saturation and capacity (veh/h) for lane *k* at arm *i*, respectively; *T*_a_ is the duration of analysis period (h), and is taken as 0.25h in this paper [28].

## 4. Solution algorithm

As the objective function and some constraints of the established model are nonlinear, and some variables are binary, the model belongs to the category of BMINLP. This section designs a genetic algorithm (GA) tailored to the characteristics of the model in order to obtain a converged solution.

### 4.1. Encoding of the solution

**Fig 5:**
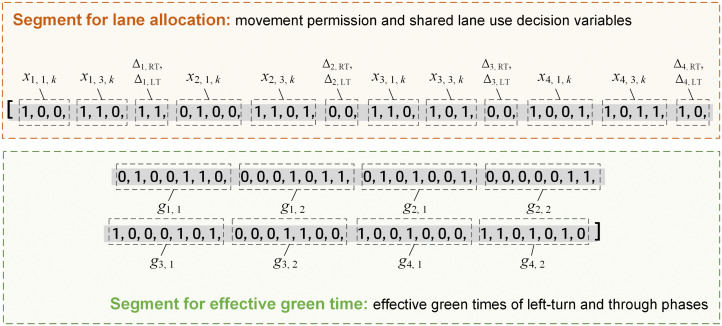
A complete example of the binary encoded solution for the model.

Every solution of the model is treated as an individual of a population, and is encoded as a binary string (gene). A complete example of the binary encoded solution is shown in Fig 5.

In Fig 5, the area with a gray background represents a complete example of the binary encoded solution, which is for a typical intersection with four arms. The string is divided into two segments. The first segment contains binary numbers for lane allocation, including decision variables related to movement permission and shared lane use at each arm, and the length is equal to 2*(*n*_1_ + *n*_2_ + *n*_3_ + *n*_4_) + 2*4 (*n*_1_ = *n*_3_ = 3 and *n*_2_ = *n*_4_ = 4 in Fig 5). It should be noticed that only left and right-turn movements are included in this segment, while the binary numbers for through movement are intended to be generated during algorithm execution.

The second segment contains binary numbers related to the effective green times of left-turn and through phases shown in Fig 2(a), and *g*_*i*, *j*_ is the effective green time for phase (*i*, *j*) (s). Assuming that each phase occupies a length of *n*_b_ (*n*_b_ = 7 in Fig 5), the length of this segment is equal to 8*n_b_. The binary numbers for right-turn phases are also to be generated during algorithm execution.

### 4.2. Algorithm design

**Fig 6:**
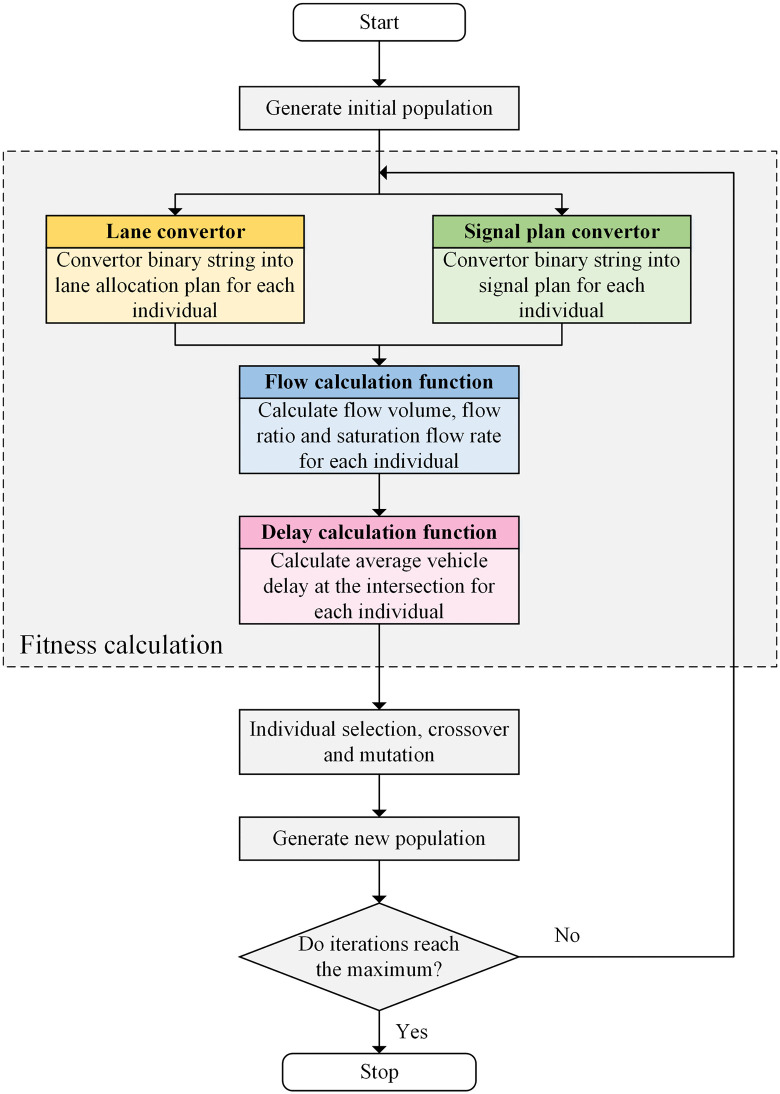
The overall process of designed genetic algorithm.

Based on the binary encoded solution, the overall process of designed GA, including initial population generation, fitness calculation and population evolution [31,32], is depicted as a flow chart in Fig 6.

**Fig 7:**
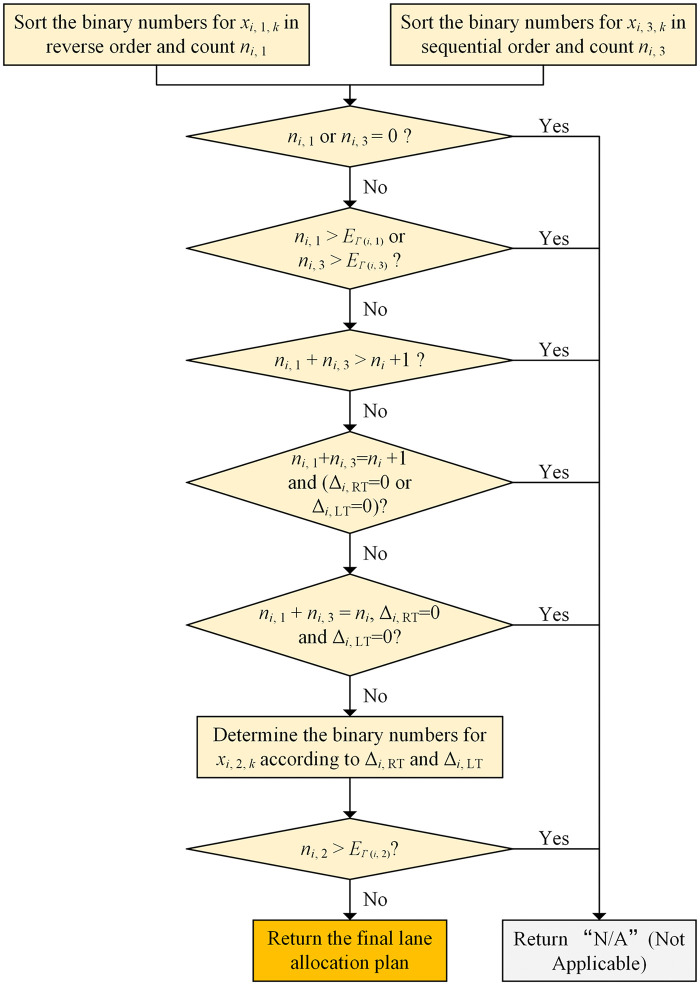
The process of the lane convertor module for each intersection arm.

Fig 6 shows that the major part of the GA is the fitness calculation, and it contains four modules named lane convertor, signal plan convertor, flow calculation function and delay calculation function. The module of lane convertor is to convert part of the binary string into the lane allocation plan for each solution (individual), and the process of this module for each intersection arm is depicted as a flow chart in Fig 7.

Fig 7 shows that the primary function of the lane convertor module is to determine whether the generated binary string can be converted into a valid lane allocation plan. If so, it returns the final lane allocation plan; if not, it outputs “N/A”. According to Fig 7, invalid lane allocations include: 1) There is no lane available for movement (*i*, *j*); 2) The total number of lanes for movement (*i*, *j*) exceeds the number of corresponding exit lanes; 3) A certain kind of shared lane needs to be allocated, but the value of the corresponding decision variable is zero.

The module of signal plan convertor is to convert part of binary string into signal control plan for each solution (individual). First, the binary string is converted into effective green time for each left-turn or through phase by using Equation (29):


$$g_{i,j}=int(g_{i,j,\min}+\frac{g_{i,j,\max}}{2^{n_{b}-1}}\cdot \sum_{v=1}^{n_{b}}b_{w,v}\cdot 2^{n_{b}-v}),\;\forall i\in A,\;j\in \{1,\;2\},\;w=2i+j-\;2$$ (29)

where *g*_*i*, *j*, min_ is the minimum effective green time for phase (*i*, *j*) (s); *g*_*i*, *j*, max_ is the maximum effective green time for phase (*i*, *j*) set in the algorithm (s); *n*_b_ is the length of binary string occupied by each phase; *b*_*w*, *v*_ is the *v*th binary number of the *w*th phase (*v*∈{1, 2, …, *n*_b_}, *w*∈{1, 2, …, 8}). After that, the durations of right-turn phases at arm *i* are determined according to Fig 2(b). If shared through-right lane is not employed at arm *i*, the right-turn phase can overlap with through one at arm *i* and compatible left-turn one at arm *Γ* (*i*, 3); Otherwise, the right-turn phase can only overlap with through one at arm *i*. This can be expressed by the follow:


$${𝛷}_{i,3}=\left\{ \;\;\;\begin{array}{c} {𝛷}_{i,2}+{𝛷}_{𝛤(i,3),1},\;\Delta_{i,RT}=0\\ {𝛷}_{i,2},\;\Delta_{i,RT}=1 \end{array}\right.\;\;\;\;\;,\;\forall i\in A$$ (30)

**Fig 8:**
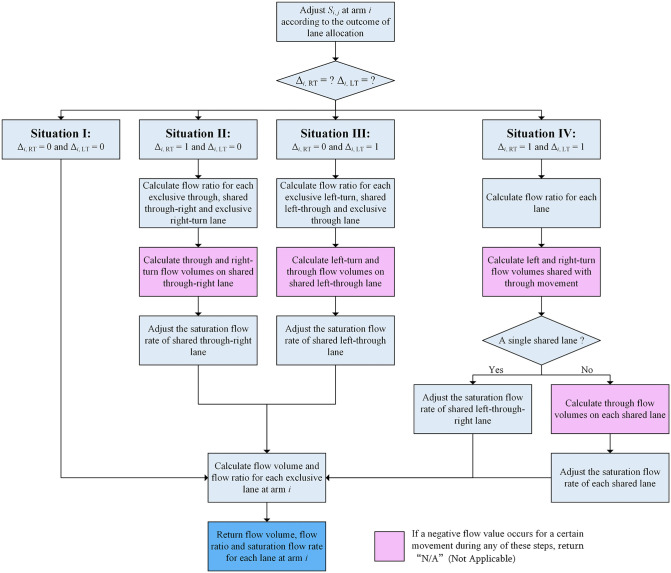
The process of the flow calculation function module for each intersection arm.

The module of flow calculation function is to calculate the flow volume, flow ratio and saturation flow rate for each approach lane at arm *i* according to the lane allocation plan generated by lane convertor module and the traffic demand at the intersection. The process of flow calculation function module is similar to that described in the section titled “*3.3.4 Flow Volume, Flow Ratio and Saturation Flow Rate*”, and is depicted as a flow chart in Fig 8.

From Fig 8, we can see that the module returns two types of values: one is the flow volume, flow ratio, and saturation flow rate for each lane at arm *i*, which is displayed in a blue box; the other is “N/A” (Not Applicable) when a negative flow value occurs for a certain movement during any of the flow volume calculation steps, and this is displayed in a pink box. The second type of value occurs when the lane configuration (generated by the lane converter module) is not compatible with the traffic flow volume.

After obtaining the flow ratio, saturation flow rate for each approach lane, and the signal control plan, the delay calculation function module is utilized to calculate average vehicle delays for each lane and the entire intersection. The calculation methods have already been presented in Equations (27) and (28). Also, if the lane convertor module returns “N/A” for a certain solution, the delay calculation function is skipped and the algorithm assigns a significantly large delay value to this solution.

## 5. Numerical examples

**Fig 9:**
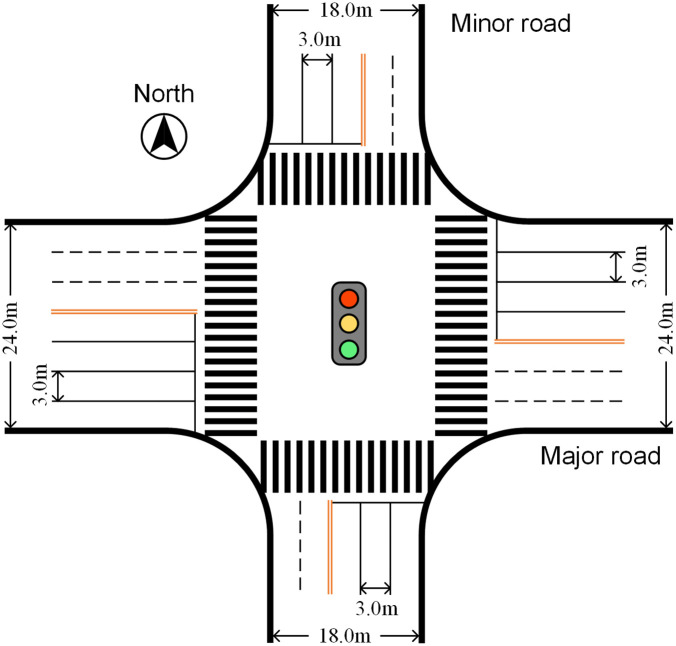
The physical layout of example intersection.

In this section, a typical intersection with four arms is designed as the basis for the numerical examples. The east-west direction is designated as the major road, while the north-south direction is the minor one. The physical layout of this intersection is shown in Fig 9. The traffic composition (heavy vehicle ratio) for each movement on each road at this intersection is presented in Table 2.

**Table 2:** The heavy vehicle ratio of example intersection.

Road classification	Heavy vehicle ratio (%)		
	Left-turn	Through	Right-turn
The major road	5	10	5
The minor road	3	6	3

Besides, it is supposed that the intersection is not located in the CBD area. According to the physical layout, traffic composition and location of designed intersection, the saturation flow rate of each movement at each arm can be determined by using the methods included in HCM. Also, the clearance interval for each phase is set to 3s [30]. The pedestrians crossing time for each through phase can be calculated according to the geometric elements of Fig 9.

To validate the broad applicability of the model and algorithm presented in this paper, six flow volume combinations have been designed, as listed in Table 3. We do not choose a real-world intersection primarily because intersections in reality exhibit very limited traffic volume patterns. Although the traffic volume at a real-world intersection varies during different time periods, some patterns occur very rarely, which means that real traffic volume cannot fully validate the model. Therefore, an intersection with a certain geometric shape is designed to closely resemble a real one, and six flow combinations are assigned to it.

**Table 3:** Six flow volume combinations for example intersection.

Arm	Movement	Flow volume (veh/h)					
		Combination Ⅰ	Combination Ⅱ	Combination Ⅲ	Combination Ⅳ	Combination Ⅴ	Combination Ⅵ
South-bound	Left-turn	130	230	20	20	350	30
	Through	360	20	330	50	400	100
	Right-turn	230	260	30	40	320	20
West-bound	Left-turn	260	560	20	70	560	150
	Through	580	50	900	150	730	320
	Right-turn	240	610	50	90	500	90
North-bound	Left-turn	150	220	30	30	370	380
	Through	310	30	310	70	420	650
	Right-turn	230	210	50	20	400	120
East-bound	Left-turn	220	600	40	120	640	830
	Through	520	30	1020	150	800	1180
	Right-turn	270	550	30	100	670	220

Each combination in Table 3 corresponds to a specific traffic distribution pattern for each movement at the arm or on the major/minor road. The pattern of each combination can be described as the follows:

▪ **Combination **Ⅰ**:** The overall traffic flow volume is moderate, with the through flow volume being larger than the left or right-turn one at each arm (approximately twice that of right-turn on the major road and 1.5 times on the minor road).
▪ **Combination **Ⅱ**:** The overall traffic flow volume is moderate, with the through flow volume being much smaller than left or right-turn one at each arm.
▪ **Combination **Ⅲ**:** The overall traffic flow volume is moderate, with the through flow volume being much larger than left or right-turn volume at each arm.
▪ **Combination **Ⅳ**:** Traffic flow volume is small at all arms, with the through flow volume being larger than left or right-turn one at each arm.
▪ **Combination **Ⅴ**:** Traffic flow volume is large at all arms, with the through flow volume being larger than left or right-turn one at each arm.
▪ **Combination **Ⅵ**:** Traffic flow is unequally distributed on the major/minor road with one arm being much larger than the other.

**Fig 10:**
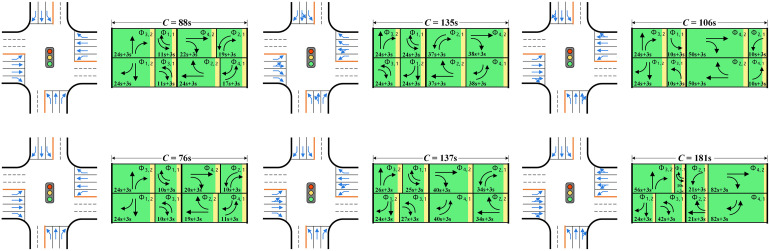
The lane allocation and signal control plans of six flow volume combinations. (a) Combination Ⅰ. (b) Combination Ⅱ. (c) Combination Ⅲ. (d) Combination Ⅳ. (e) Combination Ⅴ. (f) Combination Ⅵ.

The integrated optimization model and the designed GA are then applied to the example intersection with six flow combinations. During the execution of the algorithm, the *T*_*i*, *j*, min_ is set to 10s minimum green time plus the clearance interval for each phase (13s); The *g*_*i*, *j*, min_ is set to 10s for left-turn phases and *P*_*i*, 2_ minus the clearance interval for through phases; The *g*_*i*, *j*, max_ is set to 100s for each phase, so that the *n*_b_ is 7; The population size of GA is set to 200. During each iteration, the individual with the highest rank (the one with the smallest average vehicle delay) is directly selected for the next generation. Fifty individuals are randomly generated to maintain the diversity of solutions, while the remaining individuals undergo crossover and mutation operations, with the mutation probability set to 0.5. Finally, the lane allocation results and signal control plans of six combinations are obtained and shown in Fig 10.

Fig 10(a) shows the optimization results of Combination Ⅰ. As the through flow volume is approximately twice that of left and right turns on the major road, two through lanes are allocated for westbound and eastbound arms, compared to one lane for left and right-turn movements, respectively. On the minor road, although the through flow volume is larger than left or right-turn (approximately 1.5 times that of right-turn), shared lane use is still not the optimal solution, so that one lane is allocated for each movement. As for the signal control plan, due to the almost balanced flow volumes on either of the opposite arms, the durations of through or left-turn phases on either of the opposite arms are almost equal.

Fig 10(b) shows the optimization results of Combination Ⅱ. As through flow volume is much smaller than left or right-turn one at each arm, a shared left-through lane is employed at each arm, and no exclusive lane is provided for through movement. However, all right-turn movements are not shared with through ones and are provided with exclusive right-turn lanes. The reason is that the right-turn phase can overlap with two other phases under the condition of exclusive lane, allowing the right-turn movement to receive more green time, thereby increasing efficiency.

Fig 10(c) shows the optimization results of Combination Ⅲ. As through flow volume is much larger than left or right-turn one at each arm, a shared through-right lane is employed at each arm, and no exclusive lane is provided for right-turn movement. However, left-turn movements still operate on exclusive lanes without sharing them with through ones. This can be explained by the fact that left-turn and through movements at the same arm must operate concurrently when a shared left-through lane is used. As a result, the through vehicles on the opposite arms will be released separately, leading to an increase in green time and a loss of efficiency.

Fig 10(d) shows the optimization results for the small traffic volume combination. Under this combination, it can be seen that only extensive lanes are employed at each arm, and the duration of each phase is equal or close to the minimum green time plus the clearance interval. For this combination, the primary constraint is minimum green time instead of traffic efficiency.

Fig 10(e) shows the optimization results for the large traffic volume combination. Under this combination, shared left-through lanes are employed at each arm on the major road, which can make the lane allocation better accommodate traffic volume. Additionally, the left-turn and through movements at each arm would operate concurrently. Meanwhile, only one extensive lane is provided for each right-turn movement. This is because the right-turn phase can overlap with two other phases when an exclusive lane is used, so that more space can be allocated to left-turn and through movements.

Fig 10(f) shows the optimization results for the combination of unequally distributed traffic flow. Under such combination, both shared left-through and through-right lanes are employed at each arm on the major road. Since traffic flow is unequally distributed among the arms, the shared left-through lane allows the vehicles to be released one arm by the other, enabling two movements with high volumes to operate concurrently. As the through volume is significantly larger than the right-turn one, the shared through-right lane can provide more spatial resources for through movement. The cycle length for this combination exceeds 180s, which is longer than that of the other combinations.

**Fig 11:**
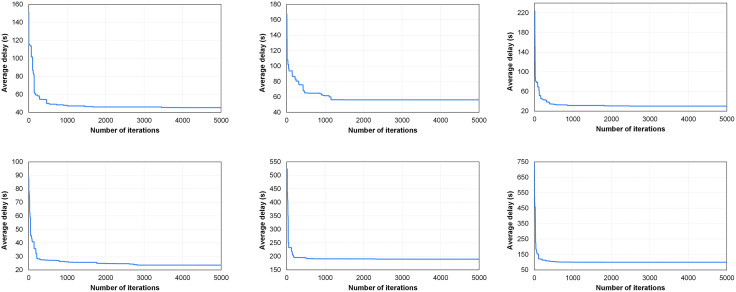
Convergent curves of the designed GA. (a) Combination Ⅰ. (b) Combination Ⅱ. (c) Combination Ⅲ. (d) Combination Ⅳ. (e) Combination Ⅴ. (f) Combination Ⅵ.

Fig 11 shows the convergent curves of the designed GA for six flow volume combinations. It can be observed that for Combinations Ⅳ, Ⅴ and Ⅵ, the GA quickly reaches a solution that has significantly minimized the average delay, and this solution is also close to the convergent one. For Combinations Ⅰ and Ⅲ, the GA obtains a significantly convergent solution when the number of iterations reaches approximately 500. For Combination Ⅱ, the average delay is significantly improved when the number of iterations reaches approximately 500 and 1100, respectively. Generally speaking, for any of the combination, the GA achieves significant convergence within 2000 generations. After that, better solutions can still be found, but the improvement is minimal. This indicates that the designed GA is suitable for solving the integrated optimization model for the IOSTRI problem proposed in this paper, and the algorithm can accommodate a wide range of traffic demands. Additionally, the computation time of the GA is recorded, taking between 40–55 seconds to reach the 2000th generation for any flow volume combination under Windows 10 environment, characterized by i5-7200U CPU, 2.71 GHz processor, and 8 GB of RAM. The computation speed presents superiority over previous studies, and is capable of meeting the requirements for real-time adaptive control of a single intersection. This is because almost all adaptive traffic signal control systems require more than 1 minute to optimize signal timing parameters. For instance, the SCOOT system typically operates every 2–10 minutes to optimize the cycle length [33].

The model and algorithm can also be applied to real-world intersections. For intersections with a fixed lane configuration (e.g., in human-driven vehicle environment). Considering the variation in traffic volume throughout the day, we can first select several typical periods (such as morning and evening peak hours, off-peak hours) and apply the model to each period. This approach allows us to obtain lane allocation and signal control plans for each period. Since the lane configuration is fixed at the intersection, we can establish a compromise lane allocation plan by comparing the plans from each period. While this compromise plan may not be optimal for every individual period, it is still satisfactory.

For intersections with variable function lanes (e.g., in connected and automated vehicle environment), lane allocation and signal control plans can be directly assigned to each period. This requires an intersection to be equipped with traffic detectors and variable lane information boards, along with the establishment of a local area network connecting the detectors, variable lane information boards, signal controller at the intersection, and the traffic control center. The detector collects the traffic flow volume at the intersection and sends it to the traffic control center. The traffic control center then predicts the traffic flow volume for future time periods, optimizes the lane allocation and signal control plans, and communicates these plans to the signal controller and the variable lane information board at the intersection. Finally, the variable lane information board and signal controller adjust the lane allocation and control plan for a specified future time period.

## 6. Conclusion

The Integrated Optimization of Spatiotemporal Resources at the Intersection (IOSTRI) is important research topic in the field of traffic signal control, and it integrates the lane allocation and signal control plans into a unified framework. The problem of IOSTRI can be categorized into two types: cycle length minimization (or capacity maximization) and delay minimization. The former is usually formulated as binary mixed integer linear program (BMILP), and the global optimal solution can be obtained by the branch and bound algorithm. The latter is typically expressed as binary mixed integer non-linear program (BMINLP). Existing research on the IOSTRI problem with delay minimization cannot obtain the optimal solution (or a near-optimal one) within a reasonable computation time. Additionally, the use of shared lanes and the lane utilization adjustment factor are not fully incorporated.

To address this problem, this paper establishes a BMINLP model for the IOSTRI problem with delay minimization as its optimization objective, and constraints that include lane allocation, lane utilization, ring-barrier structure, phase duration, flow volume, flow ratio and saturation flow rate. In the constraint regarding lane utilization, the lane utilization adjustment factor from HCM is introduced to adjust the saturation flow rate when more than one lane is provided for a specific movement. In the constraints related to flow volume, flow ratio, and saturation flow rate, four situations of lane function configuration are discussed, encompassing all possible combinations of exclusive and shared lanes. After that, a genetic algorithm (GA) tailored to the model’s characteristics is designed, where the solution is encoded as a binary string consisting of two segments representing the lane allocation and signal control plans, respectively. The main part of the GA includes four modules: lane converter, signal plan converter, flow calculation function and delay calculation function, which are used to calculate the fitness of each solution. The numerical example is based on a typical four arm intersection with six flow volume combinations. The results show that the proposed model and algorithm can adapt to various traffic flow distribution patterns. The computation time ranges from 40 to 55 seconds to obtain a high-quality solution, which represents a significant improvement over previous studies, and meets the needs for real-time adaptive control of a single intersection.

The limitation of this study is that the GA does not guarantee a global optimal solution for the integrated optimization model with delay minimization. Therefore, future studies will focus on developing algorithms that can find the global optimal solution with high computation speed. Additionally, more elements of the intersection, such as bus exclusive lanes, unconventional left-turn lanes, and access control management, will be incorporated to enhance the universality of the model.

## Supporting information

S1 File The orginal data of Fig 11. (XLSX)
